# Supplementary material for: OpDetect: A convolutional and recurrent neural network classifier for precise and sensitive operon detection from RNA-seq data
Source: PLoS One. 2025 Aug 1;20(8):e0329355. doi: 10.1371/journal.pone.0329355 (PMC12316264; doi:10.1371/journal.pone.0329355)
Supplement: S1 File — (PDF) [file pone.0329355.s001.pdf]

OpDetect's Supplementary Material

Rezvan Karaji and
Lourdes Peña-Castillo
Memorial University of Newfoundland

July 7, 2025

Contents

1 **Supplementary Tables** 2

2 **Supplementary Figures** 5

1 Supplementary Tables

Supplementary table 1: List of Python packages with their versions.

Package	Version	Package	Version	Package	Version
numpy	1.24.4	pandas	2.1.0	matplotlib	3.8.4
scipy	1.10.1	tensorflow	2.16.1	scikit_learn	1.2.1

Supplementary table 2: Machine learning summary table as per DOME recommendations (https://doi.org/10.1038/s41592-021-01205-4).

DOME	Version	1.0
**Data**	Provenance	See Table 1 in manuscript. Some data was previously used elsewhere.
	Dataset splits	10-fold cross-validation used to find the optimal hyper-parameters for the CNN-LSTM architecture.<br>See Table 2 in manuscript for number of instances per label in training data.<br>Instances per label in validation set described in Table 6 in manuscript.
	Redundancy between data splits	There is not intersection between organisms used in training and validation.<br>To make the split more strict, validation data contains data from a phylum (Spirochaetota*)* not included in the training data, and for a eukaryote (all training data is from bacterial species).
	Data availability	Yes. https://github.com/BioinformaticsLabAtMUN/OpDetect
**Optimization**	Algorithms	An ensemble of ten CNN-LSTM networks. Proposed before by Singh et al. (see complete reference in manuscript)
	Meta-predictions	No
	Data encoding	RNA-seq read counts across nucleotide bases in a genome. See section *Feature Representation* in manuscript for more details.
	Parameters	See Tables 3 and 4 in manuscript. Optimized using 10-fold cross-validation
	Features	No feature selection was performed.
	Fitting	10-fold cross-validation was used to find the optimal hyper-parameters for the CNN-LSTM model.<br>Independent validation data used to further evaluate performance.
	Regularization	Dropout and early stopping.
	Model availability	Yes. https://github.com/BioinformaticsLabAtMUN/OpDetect
**Model**	Interpretability	Black box.
	Output	Classification – probability of pair of genes belonging to the same operon.
	Execution time	In a high-performance computing environment, executing times are as followed:<br>• 10-fold CV (training) 1h23m, RAM 1.7 GB, 4 cores<br>• Prediction for ~5.6k gene-pairs requires ~3m20s, RAM 540MB and 1 core.
	Availability of Software	Yes. https://github.com/BioinformaticsLabAtMUN/OpDetect
**Evaluation**	Evaluation method	Cross-validation.<br>Independent dataset.
	Performance measures	F1-score, AUROC, recall (see Tables 7 and 8, Figs. 1-3, Supplementary figures 1-7).
	Comparison	OperonSEQer, Operon Finder, Operon-mapper and Rockhopper (see Section *Comparative assessment* in manuscript).<br>Methods selected are the most recent machine learning-based ones.
	Confidence	Confidence intervals were calculated for the cross-validation results (Table 5).<br>Performed Friedman test, and all-pairs comparisons of AUROC using several pairwise post hoc tests (i.e., Miller, Nemenyi, Siegel and Quade).
	Evaluation availability	Code to perform evaluation available at https://github.com/BioinformaticsLabAtMUN/OpDetect

Supplementary table 3: 10-fold cross-validation results of a CNN with GlobalMaxPooling2D (top) and a CNN-LSTM without an attention layer (bottom). The 90% confidence interval suggests that the model's performance metric is 90% probable to be within this range.

**Performance metric**	**Mean value**	**90% Confidence interval**
**Recall**	87.89%	[86.71%, 89.07%]
**F1-score**	88.18%	[87.01%, 89.35%]
**Accuracy**	88.86%	[87.76%, 89.96%]
**AUROC**	0.879	[0.867, 0.891]

**Performance metric**	**Mean value**	**90% Confidence interval**
**Recall**	78.44%	[72.10%, 84.77%]
**F1-score**	78.29%	[70.96%, 85.61%]
**Accuracy**	81.53%	[76.72%, 86.34%]
**AUROC**	0.784	[0.721, 0.848]

2 Supplementary Figures

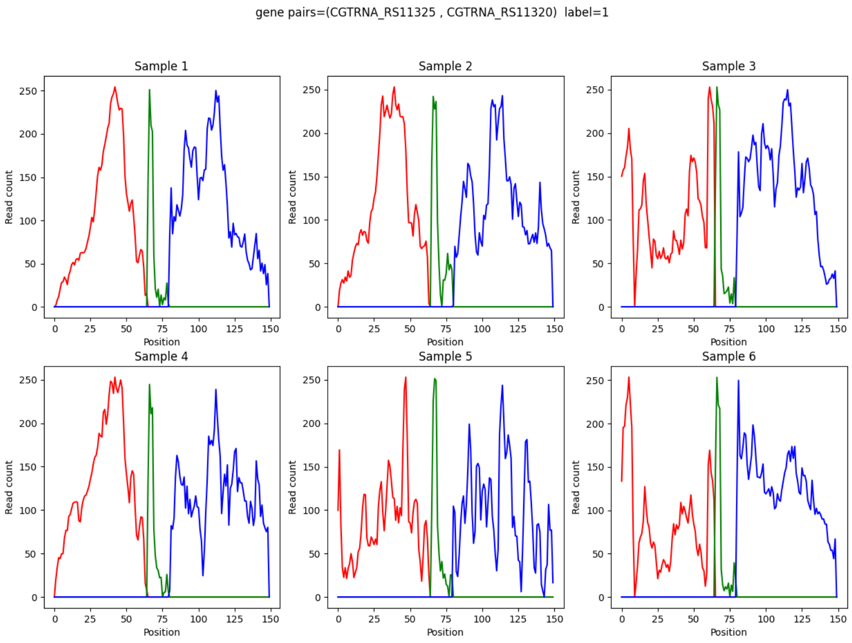

Supplementary figure 1: Feature visualization for an operon in *C. glutamicum* ATCC 13032. Each plot shows the resampled read counts for the first gene (red), intergenic region (green) and second gene (blue) for one of the six RNA-seq samples used.

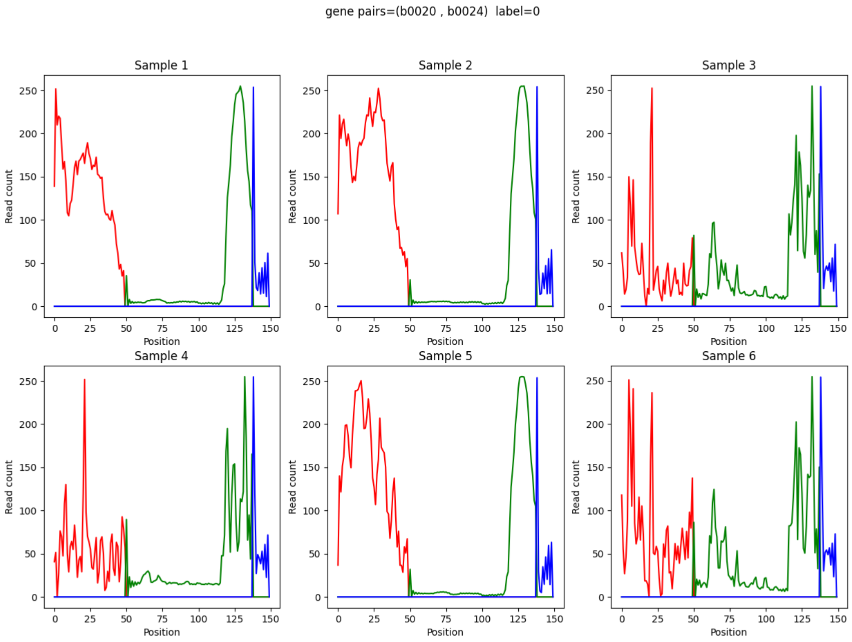

Supplementary figure 2: Feature visualization for a non-operon in *E. coli* K-12 substr. MG1655. Each plot shows the resampled read counts for the first gene (red), intergenic region (green) and second gene (blue) for one of the six RNA-seq samples used.

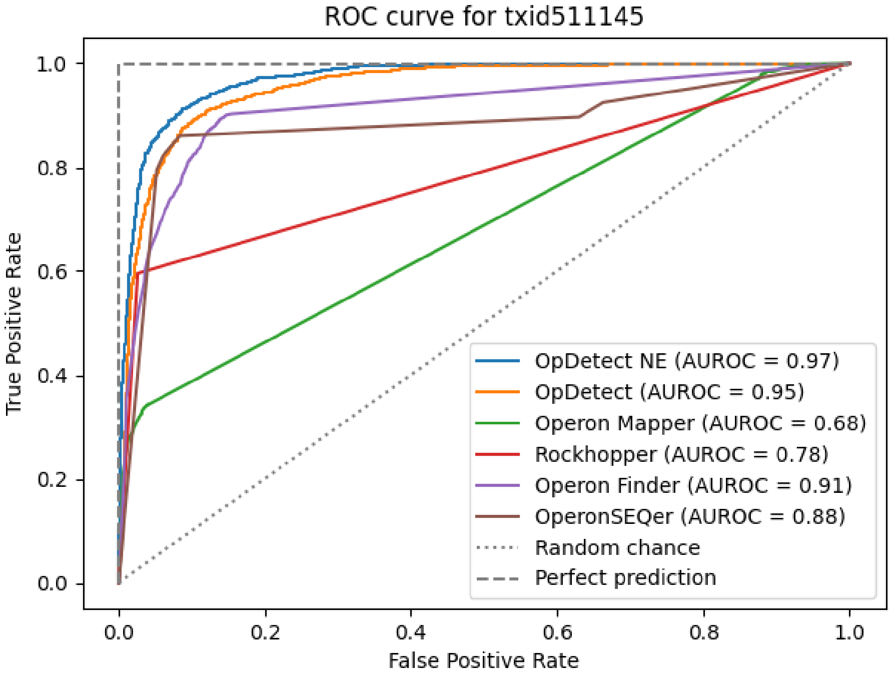

Supplementary figure 3: ROC for *E. coli* K-12 substr. MG1655. "OpDetect NE" refers to OpDetect without excluding the examined organism from the training process (i.e., affected by data leakage).

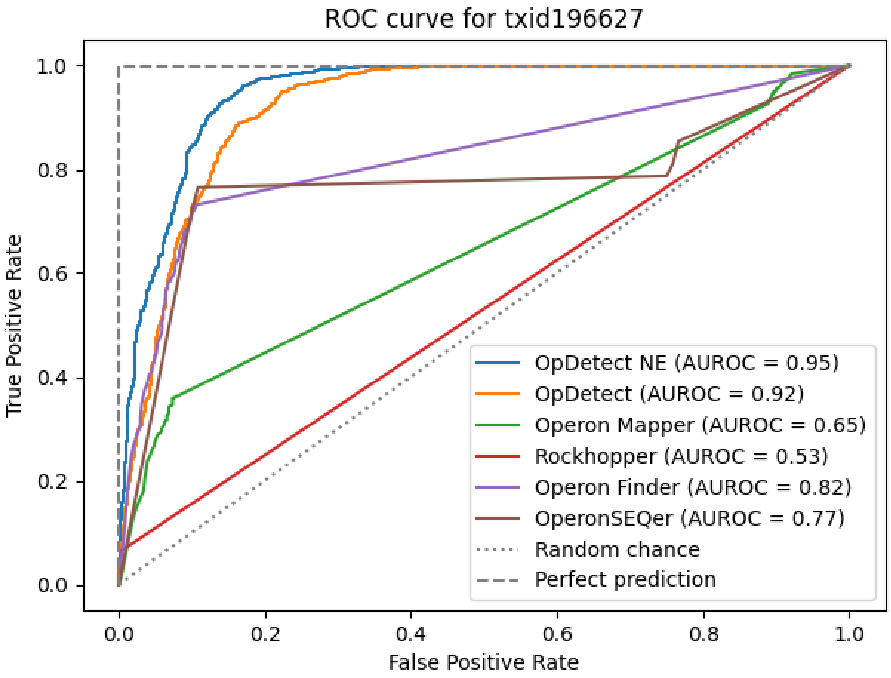

Supplementary figure 4: ROC for *C. glutamicum* ATCC 13032. "OpDetect NE" refers to OpDetect without excluding the examined organism from the training process (i.e., affected by data leakage).

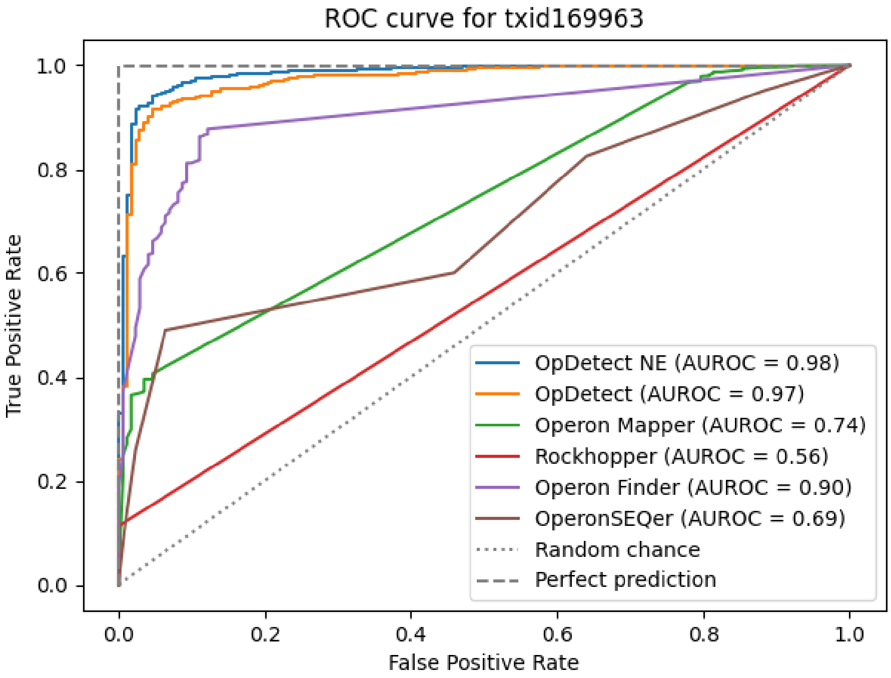

Supplementary figure 5: ROC for *L. monocytogenes* EDG-e. "OpDetect NE" refers to OpDetect without excluding the examined organism from the training process (i.e., affected by data leakage).

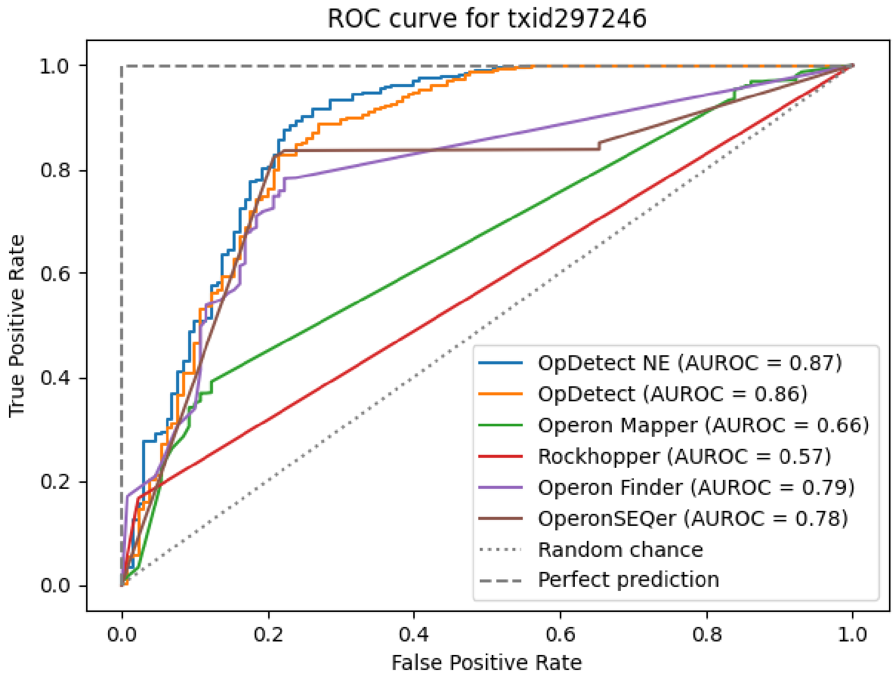

Supplementary figure 6: ROC for *L. pneumophila* str. Paris. “OpDetect NE” refers to OpDetect without excluding the examined organism from the training process (i.e., affected by data leakage).

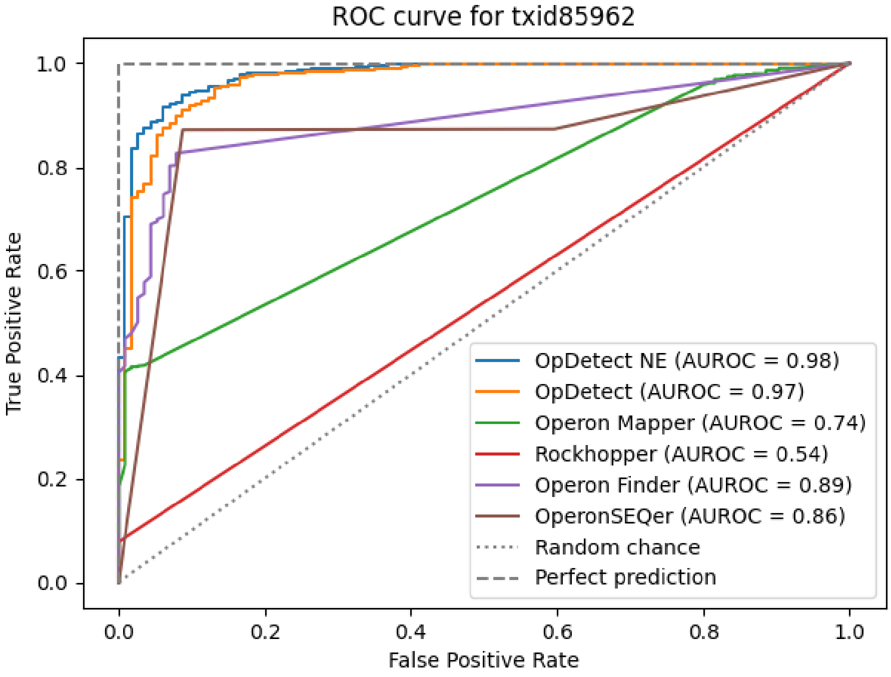

Supplementary figure 7: ROC for *H. pylori* 26695. "OpDetect NE" refers to OpDetect without excluding the examined organism from the training process (i.e., affected by data leakage).

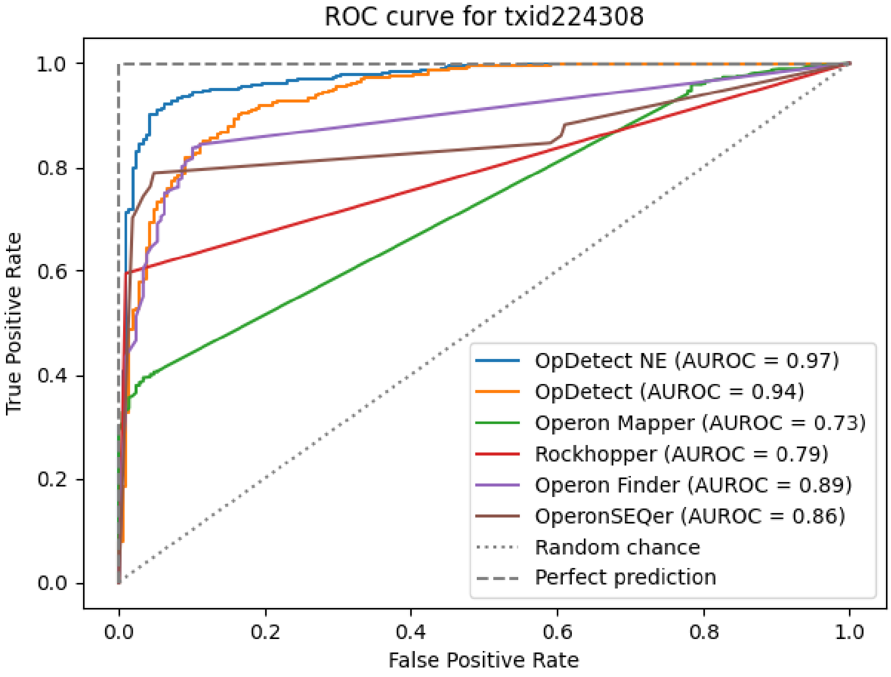

Supplementary figure 8: ROC for *B. subtilis* subsp. subtilis str. 168. "OpDetect NE" refers to OpDetect without excluding the examined organism from the training process (i.e., affected by data leakage).

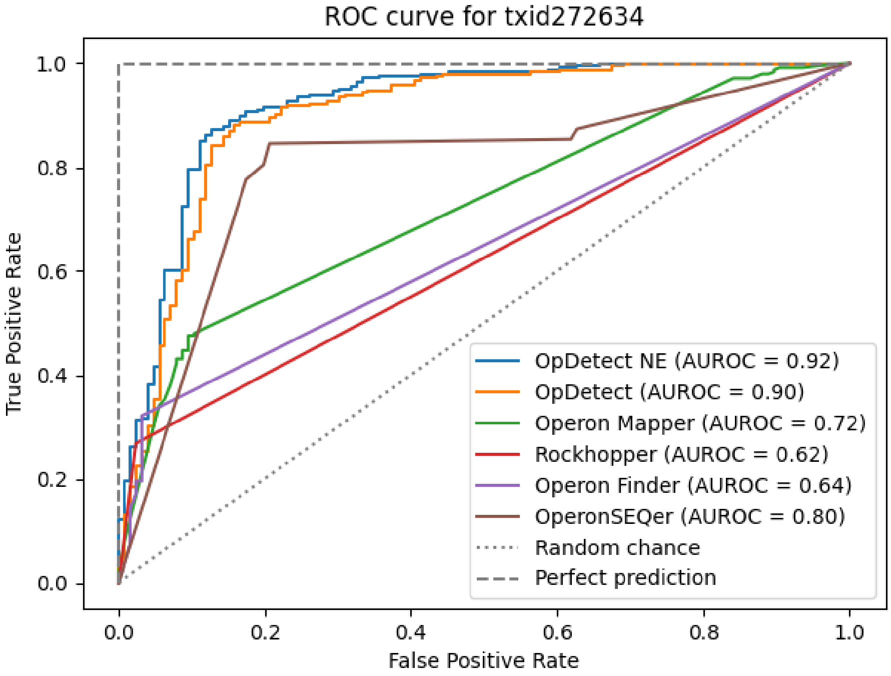

Supplementary figure 9: ROC for *M. pneumoniae* M129. "OpDetect NE" refers to OpDetect without excluding the examined organism from the training process (i.e., affected by data leakage).
