# Supplementary material for: OpDetect: A convolutional and recurrent neural network classifier for precise and sensitive operon detection from RNA-seq data
Source: PLoS One. 2025 Aug 1;20(8):e0329355. doi: 10.1371/journal.pone.0329355 (PMC12316264; doi:10.1371/journal.pone.0329355)
Supplement: Attachment — Submitted filename: OpDetect_ResponseToReviewers.pdf [file pone.0329355.s003.pdf]

Dear Dr. Petrushin,

We thank the reviewers for their time and effort reviewing our manuscript and have taken all their comments in consideration. Our answers to the reviewers' comments are in italics and bold.

Reviewer #1:

Review of OpDetect: A convolutional and recurrent neural network classifier for precise and sensitive operon detection from RNA-seq data.

Summary
The authors develop OpDetect, a deep learning approach for detecting operons in a speices-agnostic manner using RNA-sequencing (RNA-seq data). They use only genome sequences, RNA-seq data and existing operon annotation, and no additional information. They treat RNA-seq as you would signal data, and process it as such to maximize information utilization. They demonstrate that their approach outperforms state-of-the-art algorithms on recall, F1 and AUROC.
Overall, there is always the need to continually improve predictive biology programs as new data and cutting-edge algorithms come into play, and operon identification, particularly in understudied species, still requires significant research, so this work is relevant to microbiologists and computational biologists, and addresses an area of need. While the approach is distinct from anything that has been attempted and produces high metrics, a few fundamental concerns need to be addressed before publication can be recommended. Additional details are provided below, but in summary, first the authors need to justify their use of their methodology more thoroughly. Second, the metrics used are skewed by their very low numbers of 'non-operon' genes, and evaluation on such a unbalanced dataset can affect these metrics significantly. Third, the use of C. elegans data has to be justified with the biology of C. elegans operons in mind, which could change the interpretation of the data significantly.
Major points
1) The combination of two architectures to properly capture the complexity of RNA-seq data is appreciated. However, the justification is not clear. For example, did the authors try a CNN or LSTM alone and find reduced performance? The inclusion of an attention layer is interesting, but it seems like there is a missed opportunity for cross-attention or dual-attention here when asking about the relationship of RNA-seq data between two genes and an intergenic region – why the choice of self attention? Also, is the LSTM required or would more attention layers perform the same function?

As mentioned in our manuscript, we use the same architecture as described in Singh et al., 2021, since Singh et al. reported good performance on wearable sensors data that visually look like our generated features. We explored additional architectures: we tried a CNN alone, and another CNN-LSTM architecture without self-attention. We have included these results in the supplementary material and mentioned them in lines 170-177 of the manuscript. Our proposed architecture performs better than these other alternatives.

2) The small set of non-operons is a potential issue, both with training and testing. I understand the motivation behind only wanting to use experimentally validated non-operons (I assume this is the case? The authors mention the reverse case of a predicted operon being missed, but I assume the reason there are so few non-operons is because most of them are not experimentally validated to the satisfaction of the authors?). The validity of the F1 score will indeed be impacted by this as mentioned, and while using AUROC and recall are good options, they will not tell you the extent to which non-operon calls are valid

with this algorithm. It would be worth considering testing performance on putative non-operons, just to demonstrate the broader applicability of the algorithm outside of confirmed non-operons.

We used experimentally validated <u>operons</u>. Since there are no databases of experimentally validated non-operons, the restriction we have for non-operons is that both genes must be in at least one experimentally validated operon. In other words, we restricted non-operons to contiguous genes for which their corresponding operon is known. The reason for this is that if two contiguous genes have been studied and found to be in different operons, then it is more likely that they do not belong to the same operon than two contiguous genes for which their operon is unknown. This means that there are no putative non-operons unless one considers any pair of contiguous genes not known to belong to the same operon as "putative non-operons". However, this strategy of using lack of annotation as an indication that two genes do not belong to the same operon would introduce many false negatives. We have clarified this in lines 112-122.

3) The observation with C. elegans operon identification is very interesting but raises a number of concerns that the authors need to address. C. elegans operons are fundamentally biologically different from prokaryotic ones. While prokaryotic polycistronic mRNAs are not processed, but rather the genes on the mRNA are individually translated, the operon mRNAs from C. elegans are processed co-transcriptionally before translation. The other methods evaluated in this study are focused on prokaryotic operons, not eukaryotic ones, and are tailored towards detecting those.

We would like to point out that our method is also focused on prokaryotic operons and was exclusively trained on those, as already mentioned in lines 66-69.

This then leads to the question of how stably detectable C. elegans operon RNAs are, and whether the RNA preparation involved any selection (eg. polyA, which would likely preclude these intermediate RNAs, or ribo-depletion, which might preserve them though they would remain transient, or neither, which would call into question the quality of the dataset altogether). The C. elegans dataset referenced does not have this level of detail, and does not have an associated study, so clarification is needed here. As such, I wonder if another non-C.elegans eurkaryotic dataset would predict operons?

We would like to point out that the other methods' shortcoming when predicting C. elegans' operons is low recall (i.e., a high number of false negatives) instead of a high number of false positives. The test suggested by the reviewer controls for false positives. However, F1-scores and AUROC provided in Tables 7 and 8 indicate that OpDetect does not suffer from a high false positive rate. Furthermore, it has been reported that <u>at least</u> 15 to 17% of C. elegans genes are in operons [Blumenthal et al., 2002; Allen et al., 2010]. OpDetect predicts 33.75% of C. elegans genes as being part of an operon which is consistent with this known lower bound. The number of C. elegans genes predicted to be in operons by OpDetect can be considered an upper bound. These predicted operons warrant further investigation. This is discussed in lines 228-234.

And given the scarcity of non-operons and the methods used to evaluate, it's very difficult to tell whether these predictions are valid. So before claiming that this method can find the C. elegans operons, I would do a lot of due diligence with respect to:

• How the RNA from C.elegans was collected and processed, and as such are you likely to find these polycistronic mRNAs in the sample?

We rely on the description provided in the NCBI GEO database and cannot provide further information on how these data were collected and processed, since we did not generate ourselves these

data. However, OpDetect can detect these operons from expression patterns, which indicates that these polycistronic mRNAs are in the sample.

• Does the algorithm predict operons in higher organisms known not to have operons?

According to Osbourn and Field [Operons. Cell Mol Life Sci. 2009 Dec;66(23):3755-75] functional gene clusters with operon-like features have been reported in eukaryotes from yeasts to filamentous fungi, plants, and animals; and thus, selecting an appropriate eukaryotic organism to perform this test would require an extensive research on the biology of such an organism, which is outside the scope of this work.

4) The authors do not differentiate between data leakage and using the same organism for training and testing. While there is validity in training on a set of organisms and testing on a different, unseen organism, using the same organism but different datasets for training and testing is not data leakage or cross-validation. The authors should address this discrepancy and re-evaluate their assessments in the data leakage section.

We disagree with the reviewer's argument that using different datasets for the same organism is not data leakage in the context of our claims. We are following a broader understating of data leakage as described by Bernett, J., et al. [Nat Methods 21, 1444–1453 (2024)] which includes inter-sample similarities (see Question 4 of Bernett et al.) and the situation where "Test set is not drawn from the distribution of scientific interest". Our claim is that our approach can detect operons in <u>unseen</u> organisms, and thus, using the same organism for testing and training will result in overestimation of the classification performance, and, by definition, would be data leakage. We have clarified this in lines 238-239.

Minor points
1) All vectors are re-sampled to a fixed size of 150. While this is a technique used with image data, the reconstruction fidelity in RNA-seq data is not clear. Visualization of this resampled data, or some other quantitative metric to assess this would be helpful.

We have included Figures S1 and S2 in the supplementary material showing the re-sampled data for an operon and a non-operon.

2) The inclusion of a label to account for lack of annotation is interesting, but redundant (i.e. how is this different from just removing unannotated data?).

Yes, including a new label is not strictly necessary and from the point of view of training the model has the same effect as just removing unannotated data. We included this label to emphasize that data points lacking annotation might be placed into a separated category. Additionally, we can use the predictions obtained for data points with this label to guide further investigation for un-annotated operons.

3) Line 141 – I do not understand the meaning of this threshold (and it is not immediately obvious from skimming the referenced paper).

Thank you for pointing this out. We realized we were unclear in this respect. The threshold we are referring to is the confidence threshold used to classify a pair of genes as belonging to the same operon and is applied on the probabilities output by OpDetect. We have clarified this in the manuscript (lines 150-152).

4) Table 3 – why use lambda layers instead of another convolution (first lambda) or fully connected linear layer (second lambda)? Or is that lambda layer a wrapper for just that? A justification would be helpful.

The lambda layer is just used to reshape the data so that it can be fed to the LSTM layer, as mentioned in lines 130-131.

5) Table 7 – recall is an incomplete metric. I would include precision, specificity, F1 (or a subset at least) so the reader can get a better picture of overall performance.

For the organisms with enough non-operons, we have included the F1 score (upper portion of Table 7) and the ROC (Fig. 2). However, it is not possible to calculate precision, specificity or F1 score without enough non-operons (the number of non-operons per validation organism is provided in Table 6) as there are neither false positives nor true negatives. So that the reader can get a better picture of overall performance, we are also providing F1 score and AUROC for the training organisms in Table 8 (which have more non-operons, as shown in Table 2).

6) All figures were very low resolution and difficult to read.

Sorry about this. We uploaded our figures at the resolution required by the journal. We are unsure of whether the resolution was changed when inserting the figures for the reviewers' version.

7) The observation about non-contiguous genes within an operon is interesting, but not enough discussion is there on why existing programs do not identify these and OpDetect does. It is likely due to the requirement for two genes to be adjacent, not a performance issue of existing programs. If this is the case, it should be clarified.

For the other methods, we are using the provided code with their pre-trained model and what we observed and reported is that when running these other tools, they are not predicting these non-contiguous operons as operons. We neither examined their code nor retrained their model, as this is outside the scope of this work.

Also, with the gene pairs in Table 9, there should be a column stating whether the intervening antisense gene is expressed or not (i.e. is there detectable signal in the intergenic region?).

To identify these NcOs, Iturbide et al. have the restriction that the average read coverage signal at three consecutive nucleotides in the intergenic region (i.e., intervening antisense gene) does not fall below 0.1 reads per nucleotide. Thus, the intervening antisense gene is expressed. We have added this in the caption of Table 9.

Reviewer #2: The paper titled "OpDetect: A Convolutional and Recurrent Neural Network Classifier for Precise and Sensitive Operon Detection from RNA-seq Data" presents a novel, species-agnostic deep

learning approach for operon detection using RNA-seq data and sequence information.

However, a number of questions arise:

1)Could the authors clarify how they construct the gene pair vector in cases where the intergenic region is smaller than 150 base pairs?

We have added a new Fig. 1 illustrating the construction of the gene pair vector. Basically, the 150 length is for all three regions combined: gene 1, intergenic, and gene2. Only the elements in the vector that proportionally correspond to the intergenic region will have sampled values from the read counts of the intergenic region, and if there is no intergenic region, the channel for the intergenic region will be all zeros. We have clarified this in the caption of Figure 1.

2)While the architecture proposed by the authors is highly parameter-efficient and its hyperparameters are well justified, it remains an open question whether this architecture is truly optimal. Could the authors conduct an ablation study to show that modifying the number of CNN layers or removing certain components of the neural network leads to worse performance?

The potential number of alternative architectures is combinatorial and finding the optimal architecture for this task is outside the scope of our work. Nevertheless, we tried a CNN alone, and a CNN-LSTM architecture without self-attention. We have included these results in the supplementary material and mentioned them in lines 170-177 of the manuscript. Our proposed architecture performs better than these two alternatives. However, it is possible that there is an alternative architecture that might perform better than ours. We have mentioned this possibility in the manuscript, so other researchers feel encouraged to explore additional architectures.

3) I suggest that the authors report additional metrics on the set of operons that all methods were able to predict. The procedure described by the authors—where "if a method failed to make a prediction for a gene pair, we considered this as predicting that the gene pair was a non-operon and assigned a probability of 0.49"—may influence the comparison results. Clarification on how this assumption affects the evaluation would be valuable.

We argue that failing to make a prediction for a gene pair should be penalized. For example, let's assume an extreme case where a method* A *only makes one prediction and this prediction is correct, while another method* B *makes 100 predictions including the single prediction made by method* A*, but this specific prediction is incorrect. Should both methods be evaluated only on this single prediction – the set of operons that all methods were able to predict? And would this hypothetical method* A *be useful for a biologist looking to predict operons in this hypothetical organism of interest? Our answer to these two questions is no. Thus, we decided to evaluate all methods on the number of gene pairs to be predicted. Assigning a probability of 0.49 is basically indicating that this data point is on the decision boundary of the classifier (i.e., both labels are almost equiprobable) and should have less impact on the calculation of the AUROC than assigning a different probability value. We have added a discussion justifying this decision in the manuscript (lines 207-212).

4)The reformulation of the operon detection problem as a binary classification task is somewhat limiting, as it hinders the ability to capture more complex scenarios—such as when a model fails to predict some intermediate genes within a multi-gene operon, thereby compromising the full operon prediction. Could the authors comment on their choice of metric and perhaps provide counterarguments to this concern?

More complex scenarios are captured as we evaluate predictions <u>on pairs of consecutive genes</u> instead of the whole operon. Thus, the scenario the reviewer describes is already captured. That is, it is possible that OpDetect partially predicts a multi-gene operon.

5)It would also be interesting to conduct an ablation study on the features provided to the network. Is RNA-seq data alone sufficient for accurate operon prediction, or does the nucleotide sequence contribute significantly to model performance?

Thank you for this question. The nucleotide sequence is not given to the model. The model only receives the expression levels obtained from the RNA-seq data. We need the genome nucleotide sequence to map the reads to the corresponding genome and obtain the read depth coverage per base-pair. We have clarified this in the manuscript (lines 107-109).

6)If the nucleotide sequence plays an important role in prediction, could the authors investigate which parts of the sequence the model considers most informative? Are there specific motifs that the model relies on for prediction? This would be especially intriguing given the model's ability to generalize to previously unseen eukaryotic species.

As we mentioned in our answer to the previous question, nucleotide sequence data is not provided to the model. Thus, the model only "sees" expression patterns and makes predictions based exclusively on that.
